# Mathematical COVID-19 model with vaccination: a case study in Saudi Arabia

**DOI:** 10.7717/peerj-cs.959

**Published:** 2022-05-13

**Authors:** Abeer D. Algarni, Aws Ben Hamed, Monia Hamdi, Hela Elmannai, Souham Meshoul

**Affiliations:** 1 Department of Information Technology, College of Computer and Information Sciences, Princess Nourah bint Abdulrahman University, Riyadh, Saudi Arabia; 2 Sfax University, Sfax, Tunisia

**Keywords:** Mathematical model, Vaccination, Stability, COVID-19

## Abstract

The discovery of a new form of corona-viruses in December 2019, SARS-CoV-2, commonly named COVID-19, has reshaped the world. With health and economic issues at stake, scientists have been focusing on understanding the dynamics of the disease, in order to provide the governments with the best policies and strategies allowing them to reduce the span of the virus. The world has been waiting for the vaccine for more than one year. The World Health Organization (WHO) is advertising the vaccine as a safe and effective measure to fight off the virus. Saudi Arabia was the fourth country in the world to start to vaccinate its population. Even with the new simplified COVID-19 rules, the third dose is still mandatory. COVID-19 vaccines have raised many questions regarding in its efficiency and its role to reduce the number of infections. In this work, we try to answer these question and propose a new mathematical model with five compartments, including susceptible, vaccinated, infectious, asymptotic and recovered individuals. We provide theoretical results regarding the effective reproduction number, the stability of endemic equilibrium and disease free equilibrium. We provide numerical analysis of the model based on the Saudi case. Our developed model shows that the vaccine reduces the transmission rate and provides an explanation to the rise in the number of new infections immediately after the start of the vaccination campaign in Saudi Arabia.

## Introduction

The outbreak of several pandemics such as COVID-19 requires the development of mathematical models in order to exhibit key epidemiological features, investigate transmission dynamics, and develop adequate control policies. Mathematical modelling when dealing with infectious diseases allows revealing inherent patterns and underlying structures that govern outbreaks. Simple models that contain the essential components and interactions are powerful tools to test different hypotheses and understand disease control for both short and long time. The stability analysis near the free disease equilibrium will show if the apparition of new infection cases will yield to disease outbreak. Some countries such Tunisia and Jordan registered zero cases for days in Summer 2020 but the introduction of new cases resulted in critical endemic situation by Autumn.

The complex spreading patterns of COVID-19 and the various spread speed of its variants make its containing and mitigating real challenges. The existing models vary in form and complexity, but the common objective is to provide important information for global health decision makers about the disease dynamics. The first control measure was lockdown and then health authorities imposed mask wearing and social distancing. Khajanchi et al. (2021) showed, by extending the classical SEIR model, that social distance is an important factor to reduce the reproduction number and this to reduce the virus spread. Despite the herd immunity acquired *via* vaccine or infection, the social distancing is still recommended as a public health measure. Driven by the observed characteristics of COVID-19, we propose a mathematical model with two infectious states. It was reported by World Health Organization that one in three people who get COVID-19 do not show any symptoms. This is a challenging problem for health authorities as the asymptotic individuals carry the virus and may infect other people without knowing it. Moreover, consequent efforts were made worldwide since the authorisation of new vaccines by the end of 2020. By the end of November 2021, more than 50% of the world population has first dose administered and only 40% has second dose administered. In order to study the efficacy of vaccination to contain the virus spread and its negative consequences, our model include vaccinated state. The objective is to provide efficient public health policies in determining optimal vaccination strategies. Some questions have raised since the beginning of vaccination campaigns: how many individuals should be vaccinated? Is the vaccine a solution to get rid of the disease permanently? These questions are related to financial and moral costs associated with the chosen governmental policy. This paper gives theoretical and numerical analysis associated with COVID-19 epidemic dynamics in order to answer these critical questions. Although we focus mainly on the Saudi case, the model structure is general and numerically adapted to any specific context without loss of validity of the qualitative results here shown. The main contributions of this research are given as follows:

• Developing a novel mathematical model to predict the spread of COVID-19, with the presence of vaccinated and asymptotic compartments
• Analyzing the existence of endemic equilibrium point and the stability of disease-free equilibrium
• Investigating a real case study in Saudi Arabia, discussing the impact of vaccination on disease dynamics

In ‘Related Works’, we present the related works regarding epidemic modeling with a focus on COVID-19 control strategies and particularly population vaccination. ‘Proposed Model and Effective Reproduction Number’ and ‘Model Analysis’ include model description and analysis, respectively. The numerical results are given in ‘Numerical Simulations’ and ‘Conclusion’ concludes this paper.

## Related Works

The mathematical modeling in epidemiology started in England, in the 18th century, when Bernoulli analyzed the mortality of smallpox. Since then, a large variety of epidemiological models have been developed (Gumel et al., 2021; Mandal et al., 2020; Batistela et al., 2021; Samui, Mondal & Khajanchi, 2020). In this section, we present recent works proposed in this century, impacted by several outbreaks such as Ebola, Zika, and the swine flu (Bekiros & Kouloumpou, 2020). Alexander & Moghadas (2005) developed a Susceptible-Infected-Recovered-Susceptible (SIRS) epidemic model. The authors considered that the immunity acquired by the population after infection decreases over time. The dynamical behavior of the model is investigated using different types of bifurcation, including saddle–node, Hopf, and Bogdanov-Takens. The stability analysis based on the basic reproductive number and the rate of loss of natural immunity demonstrated the coexistence of two concentric limit cycles. These theoretical results have epidemiological implications such the determination of epidemic outbreak and the control the disease spread.

Yi, Zhao & Zhang (2009) investigated the Susceptible, Exposed, Infectious, Quarantine, Susceptible (SEIQS) epidemic model, with a nonlinear incidence rate. This model takes into consideration the communal sanitation measure of quarantine, aiming at avoiding broad infection. The authors provided a stability analysis using codimension-1 (transcritical, saddle–node, and Hopf) and codimension-2 bifurcations (Bogdanov-Takens).

Recently, Lu et al. (2019) studied the the SIRS epidemic model, the same considered in Alexander & Moghadas (2005) but with a generalized non-monotone incidence rate. The incidence rate is a function of the infection force of a disease and the number of susceptible individuals. The given formula for the incidence rate models the psychological pressure of some epidemic disease. The government is, in general, led to take some protective measures like lockdown when the infection number becomes very high. The authors showed that the model has both repelling and attracting Bogdanov-Takens bifurcations. Moreover, from the super-critical Hopf bifurcation, the authors concluded that a disease following this model presents periodic outbreak, which is very important to understand its dynamics, in the real world.

The impact of treatment function was investigated in Xiao et al. (2015) using the SIS model, where recovered individuals become again susceptible and the incidence rate is bi-linear. In the considered model, the treatment function is saturated, which results in the existence of backward bifurcation. Thus, the eradication of the disease is not only related to the reproduction number but also to other biological or epidemiological mechanisms, such as imperfect vaccine. The bifurcation analysis outlines the necessary conditions to eliminate the disease. Zhang, Ge & Lin (2020) discussed the impact of the number of hospital beds on SIS epidemic model, by considering a nonlinear recovery rate. The authors calculated the basic reproduction number corresponding to their model. This number determines the condition for the disease-free equilibrium to be globally asymptotically stable.

The limitations of medical resources, mainly the availability of vaccinations, is modeled using a piecewise-defined function for patient treatment in Wang, Xiao & Smith (2019). This function admits a backward bifurcation with limited available medical resources. The variation of vaccination threshold affects the existence of multiple steady states,crossing cycle, and generalized endemic equilibria. Similarly, Perez, Avila-Vales & Garcia-Almeida (2019) considered nonlinear incidence rate for a generalized SIR model. Besides, the authors assumed that the model has saturated Holling type II treatment rate and logistic growth. Non linear and saturated functions allows to represent more accurately the dynamics epidemic diseases. Similar to previous stated works, the authors revealed the importance of the basic reproduction number *R*_0_, whose value determines the existence of endemic equilibrium and the stability of the disease-free equilibrium. Under some conditions related to the disease transmission rate and the treatment rate, the model may undertake a backward bifurcation and a Hopf bifurcation. The above-mentioned articles considered general disease models. In the literature, we can also find specific models targeting particular disease such as avian influenza (Jiang et al., 2020) and bacterial meningitis (Asamoah et al., 2020). Since the declaration of World Health Organization (WHO) of the Severe Acute Respiratory Syndrome Coronavirus (SARS-CoV-2) as a pandemic on March 2020, the scientific community has been trying to understand the dynamics of this virus.

One of the measures to control the virus spread in to declare total or partial lockdown, forcing social distancing. The scientific community believes that the main cause of infection is the inhalation of virus droplets (Jayaweera et al., 2020). Gevertz et al. (2021) modeled social distancing as a flow rate between susceptible and asymptomatic individuals. The model reveals the existence of of a critical implementation delay, when implementing social distancing mandates. A delay of two weeks is the critical threshold between infection containment and infection expansion.

Nadim & Chattopadhyay (2020) investigated the effect of imperfect lockdown. In the adopted model, when the basic reproduction number, *R*_0_ is less than unity, the stable disease free equilibrium coexists with a stable endemic equilibrium. This means that COVID-19 undergoes backward bifurcation. This phenomenon was observed in the Kingdom of Saudi Arabia where the new cases were decreasing to reach 97 in 06, January 2021. Unfortunately, this rate reached 386 new cases, after one month, which obliged the Ministry of Health to declare partial lockdown for 10 days. The infection force is so high that the disease cannot be totally eradicated. The authors showed that under perfect lockdown, this backward bifurcation does not exist, but such condition is not possible in the real world. In Di Giamberardino & Iacoviello (2021), the authors included in their mathematical model, based on the classical SEIR, several prevention actions such as test campaign on the population and quarantining infected persons. The model took in consideration infection treatment efforts, such as vaccination and the therapy of induced cardio-respiratory complications. Besides the usual classes of the population, the authors considered two new classes, driven by specific characteristics of the virus: infected but asymptomatic patients and suspected infected individuals. The theoretical results, tuned using the Chinese case, were compared to United Kingdom case and the Italian case, showing the similarity between the model dynamics and the real epidemic behaviour. Lü et al. (2021) proposed an epidemic model that distinguishes between the first and the second waves od COVID-19 in China. The two-stage model includes a Contacts compartment, besides the usual Susceptible, Infectious and Recovered compartments. The authors of Khajanchi, Sarkar & Banerjee (2022) raised the issue of undetected symptomatic and asymptomatic individuals. They also investigated the effect of two control strategies that include the improvement of treatment for infected and isolated individuals. Another scientific aspect of COVID-19 is the possible transmission of the virus through contaminated surfaces. It is believed that the virus can survive several days on the surfaces depending on the material (wood, glass or plastic). Another issue faced by the governments is the awareness level of the population. Some individuals, deliberately, decide not to apply the precautionary measures, mainly wearing mask and respecting social distancing (Kassa, Njagarah & Terefe, 2020).

The issue of the efficiency of social distancing and rapid testing strategies against the pandemic was examined in Aldila et al. (2020), where the authors extended the standard SEIR model. The authors considered also the problem of undetected asymptomatic individuals, who have no symptoms but participate actively to virus spread. Furthermore, the limitation of medical resources was incorporated to the model. The theoretical findings emphasized the role of the basic reproduction number *R*_0_ in the existence of stable COVID-19 free and COVID-endemic equilibrium points. This conclusion is contested by Mohd & Sulayman (2020), who studied the SIRS model with limited medical resources and false detection issues. The authors showed that the condition of reducing the basic reproduction number under the unity value is necessary to eliminate the disease but not sufficient. Since the authorization COVID-19 vaccines, several research works focused on giving insight to mathematical characteristic of virus spread after population vaccination. Algehyne & Ibrahim (2021) used nonlinear functional analysis and fractal fractional derivative to model the evolution over time of four compartments: susceptible, infected, infected positive tested, and recovered. The Spanish case was investigated in Kumar, Erturk & Murillo-Arcila (2021) using also fractional derivatives. It is important to highlight that these works do not consider vaccinated state as a separate compartment. They rather consider that the vaccinated individuals are moved from susceptible to recovered compartment. The vaccinated individuals are considered to move also from exposed state in Wintachai & Prathom (2021).

Different mathematical tools are used by Rajaei et al. (2021) to compare the effect of vaccination with social distancing and hospitalization. The extended Kalman filter (EKF) is used for state estimation under uncertainty.

Most of the existing research works developing a relationship between infectious and asymptotic individuals focus on estimating the model parameters using actual data (Asamoah et al., 2021; Gevertz et al., 2021; Sasmita et al., 2020; Giordano et al., 2020). To the best of our knowledge, our work is the first to provide to study mathematical stability of endemic and disease free equilibrium. Most relevant works in COVID-19 mathematical modeling are compared in Table 1, in terms of disease control strategy, considered model and country of the case study.

**Table 1:** Most relevant studies in the field of COVID-19 prediction trend.

Ref.	Control Measure	Model	Country of case study
Nadim & Chattopadhyay (2020)	lockdown	SEIR+Hospitalized+Lockdown	India, Mexico, South
			Africa and Argentina
Di Giamberardino & Iacoviello (2021)	test campaign and quarantine	SEIR+ Quarantined	UK, China, Italy
Gevertz et al. (2021)	social distancing	SAIR	–
Kassa, Njagarah & Terefe (2020)	social distancing and mask	SEIR+ Asymptotic	WHO
	wearing		
Aldila et al. (2020)	social distancing and rapid	SEIR+ Asymptotic	Indonesia
	testing		
Asamoah et al. (2021)	social distancing	SEIR+Quarantined+Hospitalized	Egypt and Ghana
Gevertz et al. (2021)	social distancing	SAIR	WHO
Algehyne & Ibrahim (2021)	vaccination	SIR	Brasil
Kumar, Erturk & Murillo-Arcila (2021)	vaccination	SEIRS+Death	Spain
Rajaei et al. (2021)	vaccination	SEIR+Quarantined+Hospitalized	Canada
Our research	Vaccination	SAIR+Vaccinated	Saudi Arabia

**Figure 1:**
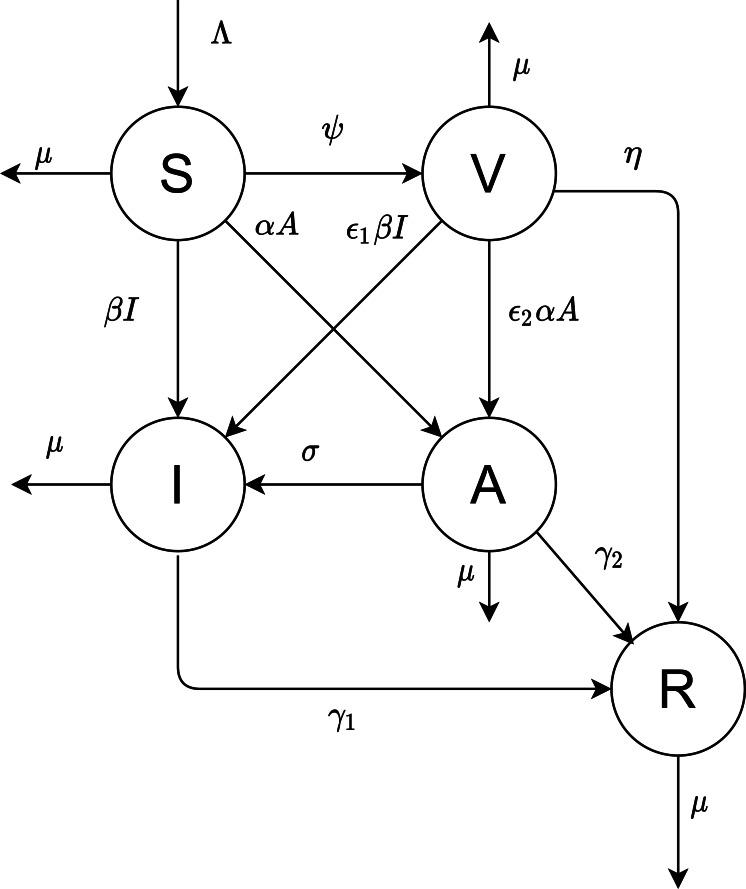
The proposed model.

## Proposed model and effective reproduction number

Our objective is to derive the mathematical equations that better present the dynamics of COVID-19 virus. The population is divided into five compartments: susceptible, vaccinated, infectious, asymptotic, and recovered; the numbers in these states are denoted by S(t), V(t), I(t), A(t), and R(t), respectively. Figure 1 depicted the flow diagram of the disease spread.

Table 2 and Table 3 summarize the different model parameters and variables.

All newborns are assumed to be susceptible. The natural recruitment and the natural death are denoted by Λ and µ, respectively. The disease-induced death rate is ignored. Susceptible individuals are vaccinated at rate constant *ψ*. The parameters *α* and *β* are the infecting rates of asymptotic and infectious individuals, respectively. *γ*_1_ and *γ*_2_ are the rates that the infectious and asymptotic individuals become recovered and acquire temporary immunity, respectively. The vaccinated individuals need a period of time to develop their immunity against the virus, represented by $ \frac{1}{\eta } $.

The virus may infect vaccinated individuals but at a lower rate than susceptible individuals who are not unvaccinated. Thus in this case, the transmission rates *β* and *α* are multiplied by a scaling factor *ɛ*_1_ and *ɛ*_2_ (0 ≤*ɛ*_1_, *ɛ*_2_ ≤1).

Based on the above assumptions and Fig. 1, we formulate the following model of differential equations.

(1a) \begin{eqnarray*} \frac{dS}{dt} & =\Lambda -\mu S-\psi S-\beta IS-\alpha AS\end{eqnarray*}

(1b) \begin{eqnarray*} \frac{dV}{dt} & =-\mu V+\psi S-\eta V-{}_{1}\beta IV-{}_{2}\alpha AV\end{eqnarray*}

(1c) \begin{eqnarray*} \frac{dI}{dt} & =-\mu I+\beta IS+{}_{1}\beta IV+\sigma A-{\gamma }_{1}I\end{eqnarray*}

(1d) \begin{eqnarray*} \frac{dA}{dt} & =-\mu A+\alpha AS-\sigma A+{}_{2}\alpha AV-{\gamma }_{2}A\end{eqnarray*}

(1e) \begin{eqnarray*} \frac{dR}{dt} & =-\mu R+\eta V+{\gamma }_{1}I+{\gamma }_{2}A\end{eqnarray*}

The basic reproduction number is defined as the number of secondary infections produced by a single infectious individual during his or her entire infectious period. Since we introduce a vaccination program in our model, it is called the effective reproduction number. The system (1) has always a disease-free equilibrium, which is obtained by setting all the derivatives to zero with I = *A* = 0, that yields to: ${P}_{0}=({S}_{0},{I}_{0},{A}_{0},{R}_{0},{V}_{0})=( \frac{\Lambda }{\mu +\psi } ,0,0, \frac{\eta \psi \Lambda }{\mu (\mu +\psi )(\mu +\eta )} , \frac{\psi \Lambda }{(\mu +\psi )(\mu +\eta )} )$

Let *x* = (*I*, *A*, *V*, *R*, *S*)^*T*^. System (1) can be rewritten as ${x}^{&prime; }=\mathcal{F}(x)&minus; \mathcal{N}(x),$, where $\mathcal{F}$ be the rate of appearance of new infections in each compartment. The progression from A to I is not considered to be new infection, but rather the progression of an infected individual through various infectious compartments.

$\begin{array}{@{}l@{}} \displaystyle \mathcal{F}(x)= \left( {\scriptsize \begin{array}{@{}c@{}} \displaystyle \beta IS+{}_{1}\beta IV\\ \displaystyle \alpha AS+{}_{2}\alpha AV\\ \displaystyle 0\\ \displaystyle 0\\ \displaystyle 0 \end{array}} \right) , \mathcal{N}(x)= \left( {\scriptsize \begin{array}{@{}c@{}} \displaystyle (\mu +{\gamma }_{1})I-\sigma A\\ \displaystyle (\mu +\sigma +{\gamma }_{2})A\\ \displaystyle (\mu +\eta )V-\psi S+{}_{1}\beta IV+{}_{2}\alpha AV\\ \displaystyle \mu R-\eta V-{\gamma }_{1}I-{\gamma }_{2}A\\ \displaystyle -\Lambda +(\mu +\psi )S+\beta IS+\alpha AS \end{array}} \right) . \end{array}$

The infected compartments are A and I, giving *m* = 2. With A =I =0, the Jacobian matrices of $\mathcal{F}(x)$ and $\mathcal{N}(x)$ at the disease-free equilibrium *P*_0_ are, respectively,

$\begin{array}{@{}l@{}} \displaystyle D\mathcal{F}({P}_{0})= \left( {\scriptsize \begin{array}{@{}cccc@{}} \displaystyle F&\displaystyle 0&\displaystyle 0&\displaystyle 0\\ \displaystyle 0&\displaystyle 0&\displaystyle 0&\displaystyle 0\\ \displaystyle 0&\displaystyle 0&\displaystyle 0&\displaystyle 0\\ \displaystyle 0&\displaystyle 0&\displaystyle 0&\displaystyle 0 \end{array}} \right) , D\mathcal{N}({P}_{0})= \left( {\scriptsize \begin{array}{@{}cccc@{}} \displaystyle N&\displaystyle 0&\displaystyle 0&\displaystyle 0\\ \displaystyle {}_{1}\beta {V}_{0} {}_{2}\alpha {V}_{0}&\displaystyle \mu +\eta &\displaystyle 0&\displaystyle -\psi \\ \displaystyle -{\gamma }_{1} -{\gamma }_{2}&\displaystyle -\eta &\displaystyle \mu &\displaystyle 0\\ \displaystyle \beta {S}_{0}   \alpha {S}_{0}&\displaystyle 0&\displaystyle 0&\displaystyle \mu +\psi \end{array}} \right) , \end{array}$

$\begin{array}{@{}l@{}} \displaystyle F= \left( {\scriptsize \begin{array}{@{}cc@{}} \displaystyle \beta {S}_{0}+{}_{1}\beta {V}_{0}&\displaystyle  0\\ \displaystyle 0&\displaystyle  \alpha {S}_{0}+{}_{2}\alpha {V}_{0} \end{array}} \right) , N= \left( {\scriptsize \begin{array}{@{}cc@{}} \displaystyle \mu +{\gamma }_{1}&\displaystyle -\sigma \\ \displaystyle 0&\displaystyle \mu +\sigma +{\gamma }_{2} \end{array}} \right) . \end{array}$

Our developed model is similar to the two-strain model in Van den Driessche & Watmough (2002) with two infectious compartments. *FN*^−1^, the next generation matrix of system (1) has the two eigenvalues.

${R}_{1}= \frac{\beta ({S}_{0}+{}_{1}{V}_{0})}{\mu +{\gamma }_{1}} = \frac{\beta \Lambda ( \frac{1}{\mu +\psi } +{}_{1} \frac{\psi }{(\mu +\psi )(\mu +\eta )} )}{\mu +{\gamma }_{1}} = \frac{\beta \Lambda (1+{}_{1} \frac{\psi }{\mu +\eta } )}{(\mu +{\gamma }_{1})(\mu +\psi )} $

${R}_{2}= \frac{\alpha ({S}_{0}+{}_{2}{V}_{0})}{\mu +{\gamma }_{2}+\sigma } = \frac{\alpha \Lambda (1+{}_{2} \frac{\psi }{\mu +\eta } )}{(\mu +{\gamma }_{2}+\sigma )(\mu +\psi )} = \frac{\alpha \Lambda (\mu +\eta +{}_{2}\psi )}{(\mu +{\gamma }_{2}+\sigma )(\mu +\psi )(\mu +\eta )} $

The effective reproduction number for the system is the maximum of the two.

**Table 2:** Model parameters and description.

Parameter	Description
Λ	Recruitment rate of susceptible humans
µ	Natural mortality rate
1/*η*	Immunity development period
*γ*_1_	Recovery rate Infectious
*γ*_2_	Recovery rate Asymptotic
1/ *σ*	Period for asymptotic individuals to develop symptoms

**Table 3:** Model variables and description.

Variable	Description
*β*	Transmission rate for Infectious
*α*	Transmission rate for Asymptotic
*ψ*	Vaccination coverage rate

## Model analysis

### Existence of endemic equilibrium point

In this section, we investigate the conditions for the existence of endemic equilibria of system (1). Any equilibrium satisfies the following equations:

(2a) \begin{eqnarray*}\Lambda -\mu S-\psi S-\beta IS-\alpha AS=0\end{eqnarray*}

(2b) \begin{eqnarray*}-\mu V+\psi S-\eta V-{}_{1}\beta IV-{}_{2}\alpha AV=0\end{eqnarray*}

(2c) \begin{eqnarray*}-\mu I+\beta IS+{}_{1}\beta IV+\sigma A-{\gamma }_{1}I=0\end{eqnarray*}

(2d) \begin{eqnarray*}-\mu A+\alpha AS-\sigma A+{}_{2}\alpha AV-{\gamma }_{2}A=0\end{eqnarray*}

(2e) \begin{eqnarray*}-\mu R+\eta V+{\gamma }_{1}I+{\gamma }_{2}A=0\end{eqnarray*}

Eq. (2d) gives the following expression:

$S= \frac{\mu +\sigma +{\gamma }_{2}}{\alpha } -{}_{2}V$.

Eq. (2b) gives the following expression:

$V= \frac{\psi (\mu +\sigma +{\gamma }_{2})}{\alpha (\mu +\eta +{}_{1}\beta I+{}_{2}\alpha A+\psi {}_{2})} $.

From Eq. (2c) and assuming *ɛ*_1_ − *ɛ*_2_ = 0, we deduce the following expressions:

$A=( \frac{\mu +{\gamma }_{1}}{\sigma } - \frac{\beta (\mu +\sigma +{\gamma }_{2})}{\alpha \sigma } )I=DI$

$S= \frac{\mu +\sigma +{\gamma }_{2}}{\alpha } \frac{(\mu +\eta +{}_{1}\beta I+{}_{2}\alpha A)}{(\mu +\eta +{}_{1}\beta I+{}_{2}\alpha A+\psi {}_{2})} $

Eq. (2a) gives the following expression:

$ \frac{\mu +\sigma +{\gamma }_{2}}{\alpha } \frac{(\mu +\eta +{}_{1}\beta I+{}_{2}\alpha DI)}{(\mu +\eta +{}_{1}\beta I+{}_{2}\alpha DI+\psi {}_{2})} [\mu +\psi +(\beta +\alpha D)I]=\Lambda $

We arrange the previous expression to get the following:

$({}_{1}\beta +{}_{2}\alpha D)(\beta +\alpha D){I}^{2}+[(\mu +\eta )(\beta +\alpha D)+(\mu +\psi )({}_{1}\beta +{}_{2}\alpha D)-\Lambda \alpha \frac{{}_{1}\beta +{}_{2}\alpha D}{\mu +\sigma +{\gamma }_{2}} ]I+(\mu +\eta )(\mu +\psi )-\Lambda \alpha \frac{\mu +\eta +\psi {}_{2}}{\mu +\sigma +{\gamma }_{2}} $

We denote by:

a = $({}_{1}\beta +{}_{2}\alpha ( \frac{\mu +{\gamma }_{1}}{\sigma } - \frac{\beta (\mu +\sigma +{\gamma }_{2})}{\alpha \sigma } ))(\beta +\alpha ( \frac{\mu +{\gamma }_{1}}{\sigma } - \frac{\beta (\mu +\sigma +{\gamma }_{2})}{\alpha \sigma } ))$

b = $[(\mu +\eta )(\beta +\alpha ( \frac{\mu +{\gamma }_{1}}{\sigma } - \frac{\beta (\mu +\sigma +{\gamma }_{2})}{\alpha \sigma } ))+(\mu +\psi )({}_{1}\beta +{}_{2}\alpha ( \frac{\mu +{\gamma }_{1}}{\sigma } - \frac{\beta (\mu +\sigma +{\gamma }_{2})}{\alpha \sigma } ))-\Lambda \alpha \frac{{}_{1}\beta +{}_{2}\alpha ( \frac{\mu +{\gamma }_{1}}{\sigma } - \frac{\beta (\mu +\sigma +{\gamma }_{2})}{\alpha \sigma } )}{\mu +\sigma +{\gamma }_{2}} ]$

c = $(\mu +\eta )(\mu +\psi )-\Lambda \alpha \frac{\mu +\eta +\psi {}_{2}}{\mu +\sigma +{\gamma }_{2}} =(\mu +\eta )(\mu +\psi )(1-{R}_{2})$

The existence of endemic equilibrium is determined by the existence of positive solutions of the quadratic equation

(3) \begin{eqnarray*}P(I)=a{I}^{2}+bI+c=0.\end{eqnarray*}

The number of endemic equilibria of the considered system depends on parameter values *a*, *b*, and *c*. This equation may have zero, one or two solutions. We denote ${R}_{20}= \frac{\alpha \Lambda }{(\mu +{\gamma }_{2}+\sigma )(\mu +\eta )} $ then ${R}_{2}={R}_{20} \frac{\mu +\eta +{}_{2}\psi }{\mu +\psi } $

We denote by ${\psi }_{crit}={}^{def} \frac{({R}_{20}-1)\mu +{R}_{20}\eta }{1-{}_{2}{R}_{20}} .$, where *R*_2_(*ψ*_*crit*_) = 1,

Since the model parameters A and I are positive, it follows that *D* > 0 and *a* > 0. Furthermore, if *R*_2_ > 1, then *c* < 0. Since $ \frac{d{R}_{2}}{d\psi } =-{R}_{20} \frac{\eta +(1-{}_{2})\mu }{(\mu +\psi )^{2}} \lt 0$ Thus, *R*_2_ is decreasing function of *ψ* and if *ψ* < *ψ*_*crit*_ , then *R*_2_ > 1. We deduce that for *R*_2_ > 1, P(I) has a unique positive root.

If *R*_2_ < 1, we have *c* > 0 and *ψ* ≥ *ψ*_*crit*_. Since *b*(*ψ*) is an increasing function of *ψ*, if *b*(*ψ*_*crit*_) ≥ 0, then *b*(*ψ*) > 0 for *ψ* > *ψ*_*crit*_. In this case, P(I) has no positive real root and the system have no endemic equilibrium.

We consider now the case where *b*(*ψ*_*crit*_) < 0. We denote by Δ(*ψ*)=^*def*^*b*^2^(*ψ*) − 4*ac*(*ψ*). If *c*(*ψ*_*crit*_) = 0, Δ(*ψ*_*crit*_) > 0. Since *b*(*ψ*) is an increasing linear function of *ψ*, there is a unique $\bar {\bar {\psi }}\gt {\psi }_{crit}$ such that $b(\bar {\bar {\psi }})=0$. and Δ(*ψ*) has a unique root $\bar {\psi }$ in $[{\psi }_{crit},~\bar {\bar {\psi }}]$.

P(I) has two roots and the system (1) has two endemic equilibria for ${\psi }_{crit}\lt \psi \lt \bar {\psi }$. and P(I) ha no real positive root and the system (1) has no endemic equilibria for $\psi \gt \bar {\psi }$.

If *R*_2_ = 1, we have *c* = 0. In this case, system has a unique endemic equilibrium for *b*(*ψ*) < 0 and no endemic equilibrium for *b*(*ψ*) > 0.

### Stability of disease-free equilibrium

The Jacobian matrix with respect to the system (1) is given by:

${J}_{0}({P}_{0})= \left[ {\scriptsize \begin{array}{@{}ccccc@{}} \displaystyle -(\mu +{\gamma }_{1})+\beta ({S}_{0}+{}_{1}{V}_{0})&\displaystyle \sigma &\displaystyle 0&\displaystyle 0&\displaystyle 0\\ \displaystyle 0&\displaystyle -(\mu +\sigma +{\gamma }_{2})+\alpha ({S}_{0}+{}_{2}{V}_{0})&\displaystyle 0&\displaystyle 0&\displaystyle 0\\ \displaystyle -{}_{1}\beta {V}_{0}&\displaystyle -{}_{2}\alpha {V}_{0}&\displaystyle -(\mu +\eta )&\displaystyle 0&\displaystyle \psi \\ \displaystyle {\gamma }_{1}&\displaystyle {\gamma }_{2}&\displaystyle \eta &\displaystyle -\mu &\displaystyle 0\\ \displaystyle -\beta {S}_{0}&\displaystyle -\alpha {S}_{0}&\displaystyle 0&\displaystyle 0&\displaystyle -(\mu +\psi ) \end{array}} \right] .$

$ \left\vert \right. \lambda -{J}_{0}({P}_{0}) \left\vert \right. =0.$

The characteristic polynomial of the Jacobian matrix at DFE is given by *det*(*J*_0_ − *λI*) = 0, where *λ* is the eigenvalue and I is 5 × 5 identity matrix. Thus, *J*_0_ has eigenvalues given by:

*λ*_1_ =  − *μ*

*λ*_2_ =  − (*μ* + *η*)

*λ*_3_ =  − (*μ* + *ψ*)

*λ*_4_ =  − (*μ* + *γ*_1_) + *β*(*S*_0_ + *ɛ*_1_*V*_0_) = (*μ* + *γ*_1_)(*R*_1_ − 1)

*λ*_5_ =  − (*μ* + *σ* + *γ*_2_) + *α*(*S*_0_ + *ɛ*_2_*V*_0_) = (*μ* + *σ* + *γ*_2_)(*R*_2_ − 1)

All the eigenvalues are strictly negative except for *λ*_4_ and *λ*_5_. These eigenvalues depend the sign of (*R*_2_ − 1) and (*R*_1_ − 1). The stability of the DFE represents the dynamics of disease free population when a small number of infected individuals introduced. Did the system stay disease free or an endemic state may appear?

Theorem 1 *Based on the Theorem of Van den Driessche & Watmough (2002), we have the following results. If R*_1_ > 1* or/and R*_2_ > 1*, then λ*_4_* or/and λ*_5_* is/are strictly positive. In this case the DFE is unstable. If R*_1_ < 1* and R*_2_ < 1*, then λ*_4_* and λ*_5_* are strictly negative. The system is locally asymptotically stable.*

## Numerical simulations

In this paper, we focus on vaccination analysis in Saudi Arabia. The presented numerical simulations provide also general results that can be applied to any region. The data set is provided by King Abdullah Petroleum Studies and Research Center (KAPSARC). It includes five classes: Tested, Cases, Recoveries, Critical, Mortalities and Active and it spans the period from 04/03/2020 to 08/11/2021. It includes also important events and measures such as international flights suspension and lockdown. We used the Simulink Tool in order to simulate different scenarios.

The death and birth rate for Saudi Arabia are estimated to be equal to 3.39 and 14.56 for 1,000 per year, respectively. The vaccination campaign started on 18/12/2020 with a vaccination coverage of the total population of 0.02% to reach about 65% of the adult population fully vaccinated in November 2021. The vaccination rate is considered a the percentage of the total population that get vaccinated per day. With approximately 45,000 administrated doses per day and a total population of 35339000 in 2021, this rate is about 0.00 127. Is this rate enough to eradicate the disease? This what we are trying to answer is this work.

The estimation of model parameters is very important to have accurate numerical results. One approach consists in calibrating the model by fitting it with reported data using, for example, least square method (Rai et al., 2022). In our paper, we used scientific reported facts about COVID-19 transmission mechanisms. The research report (Nogrady, 2020) provides information about asymptotic individuals for COVID-19. Most people, with no symptoms at the beginning, develop symptoms in 7–13 days, which corresponds to the *σ*^−1^. Recall that *γ*_1_ is the recovery rate of infectious individuals. Interpreted as the expected value of a Poisson process, *γ*_1_ can be related to the needed time from the beginning of the infection till recovery (Gevertz et al., 2021). With average recovery duration equal to 10 days (Chae et al., 2020), the recovery rate of infectious individuals is *γ*_1_ = 0.1.

Let *ω* denote the fraction of asymptotic individuals among positive cases. According to Nogrady (2020), and based on 13 studies involving 21,708 people in 2020, *ω* = 0.17. Using the same methodology as in Gevertz et al. (2021) ${\gamma }_{2}= \frac{\omega }{1-\omega } \sigma \approx 0.2\sigma $. The asymptotic people are estimated to be 42% less contagious than symptomatic individuals (Nogrady, 2020). Thus, *α* = 0.42*β*. Table 4 summarizes selected values for the model parameters.

**Table 4:** Model parameters and values.

Parameter	Range
Λ	14.56 per 1,000 per year
*β*	[0.233, 0.462] Chae et al. (2020)
*α*	0.42 *β*
*ψ*	model parameter
µ	3.39 per 1,000 per year
1/*η*	14 days
*γ*_1_	0.1
*γ*_2_	0.2 *σ*
1/*σ*	[7,13] days

By 18/12/2020, considered as time 0 in this model, the number of recoveries is equal to 351722, the number of active cases is equal to 3,014. Assuming that *ω* = 0.17, the number of initial infectious with and without symptoms is equal to 2,501 and 513, respectively.

**Figure 2:**
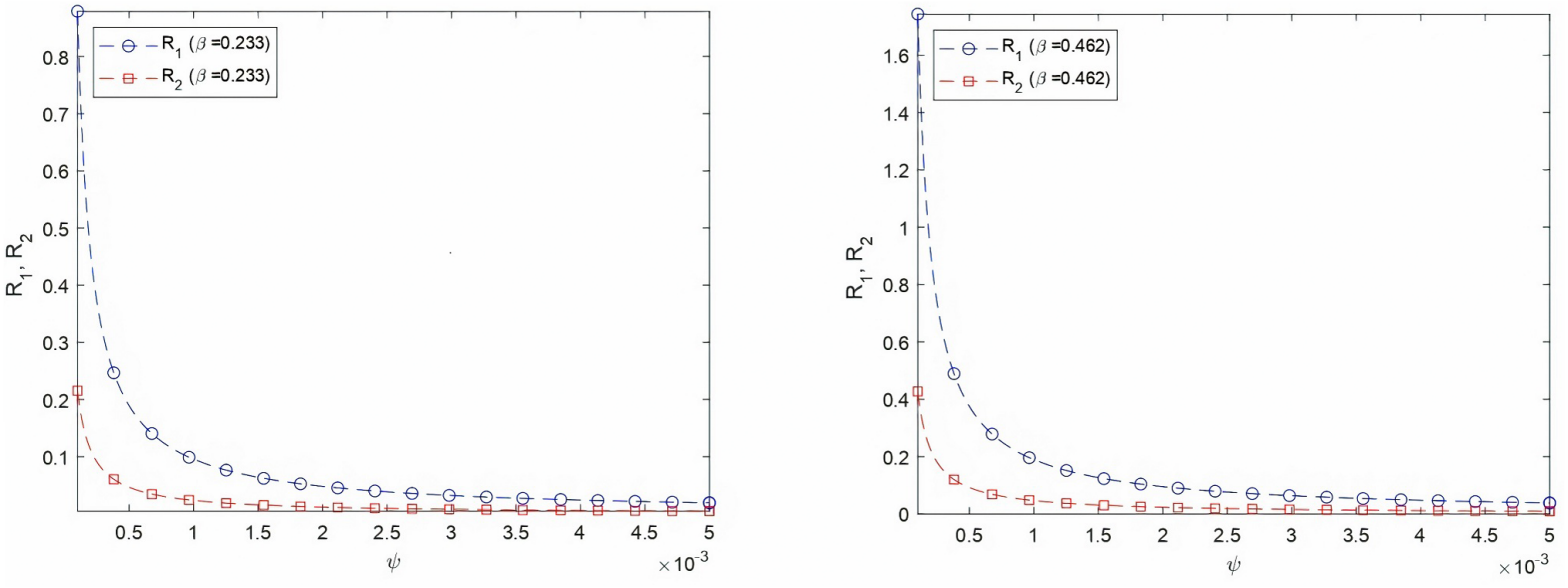
Varying *R*_1_ and *R*_2_ as a function of vaccination rate for two virus transmission rates, *β* = 0.233 and *β* = 0.462. (A) Varying *R*_1_ and *R*_2_ as a function of *ψ*, *β* = 0.233 (B) Varying *R*_1_ and *R*_2_ as a function of *ψ*, *β* = 0.462

First, we investigate the effect of the vaccination rate on the effective reproduction number defined as the maximum of the two entities *R*_1_ and *R*_2_. According to Chae et al. (2020), COVID-19 transmission rate *β* ranges between 0.233 and 0.462. Figures 2A and 2B show the evolution of both *R*_1_ and *R*_2_ as a function of the vaccination rate *ψ* with virus transmission rate equal to 0.233 and 0.462, respectively. In both cases, *R*_1_ corresponding to the strain of infectious individuals with symptoms is greater than *R*_2_, corresponding to the strain of asymptotic individuals. Thus the number of individuals infected by one person currying the virus is mainly affected by individuals showing usual symptoms. Mathematical theoretical result confirms that the vaccine reduces the spread of the virus among the population. We would like to highlight the fact in our model that a vaccinated individual may be infectious with or without symptoms. This result is very important as, till the end of 2021, an important portion of worldwide population is still opposed to vaccine. In the case of high transmission rate and low vaccination rate, *R*_1_ is higher than 1. The disease free equilibrium is consequently unstable according to theorem 1. For the Saudi case, when *beta* is equal to 0.233, *R*_1_ and *R*_2_ are equal to 0.0797 and 0.0195, respectively. When *beta* is equal to 0.462, *R*_1_ and *R*_2_ are equal to 0.1580 and 0.0387, respectively. For Saudi Arabia, the effective reproduction number is less than 1, even for high transmission rate. This result is explained by the high vaccination rate.

**Figure 3:**
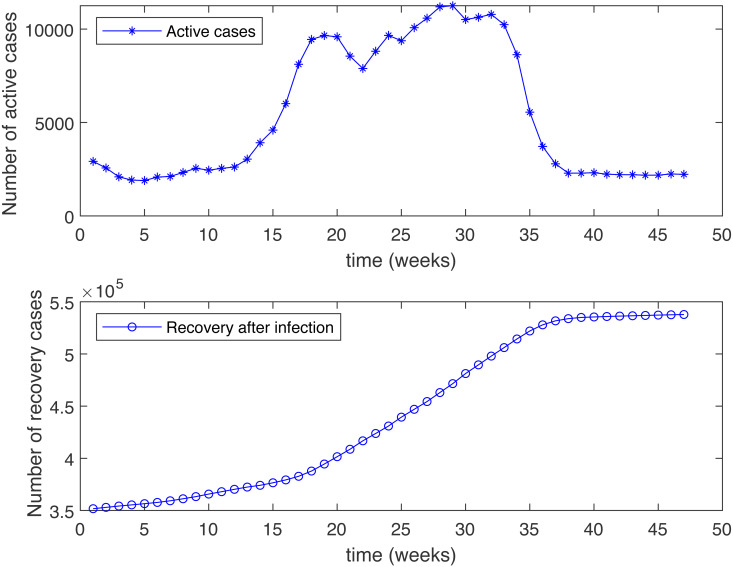
Number of active cases and recovered after infection in KSA, starting from 18/12/2020.

**Figure 4:**
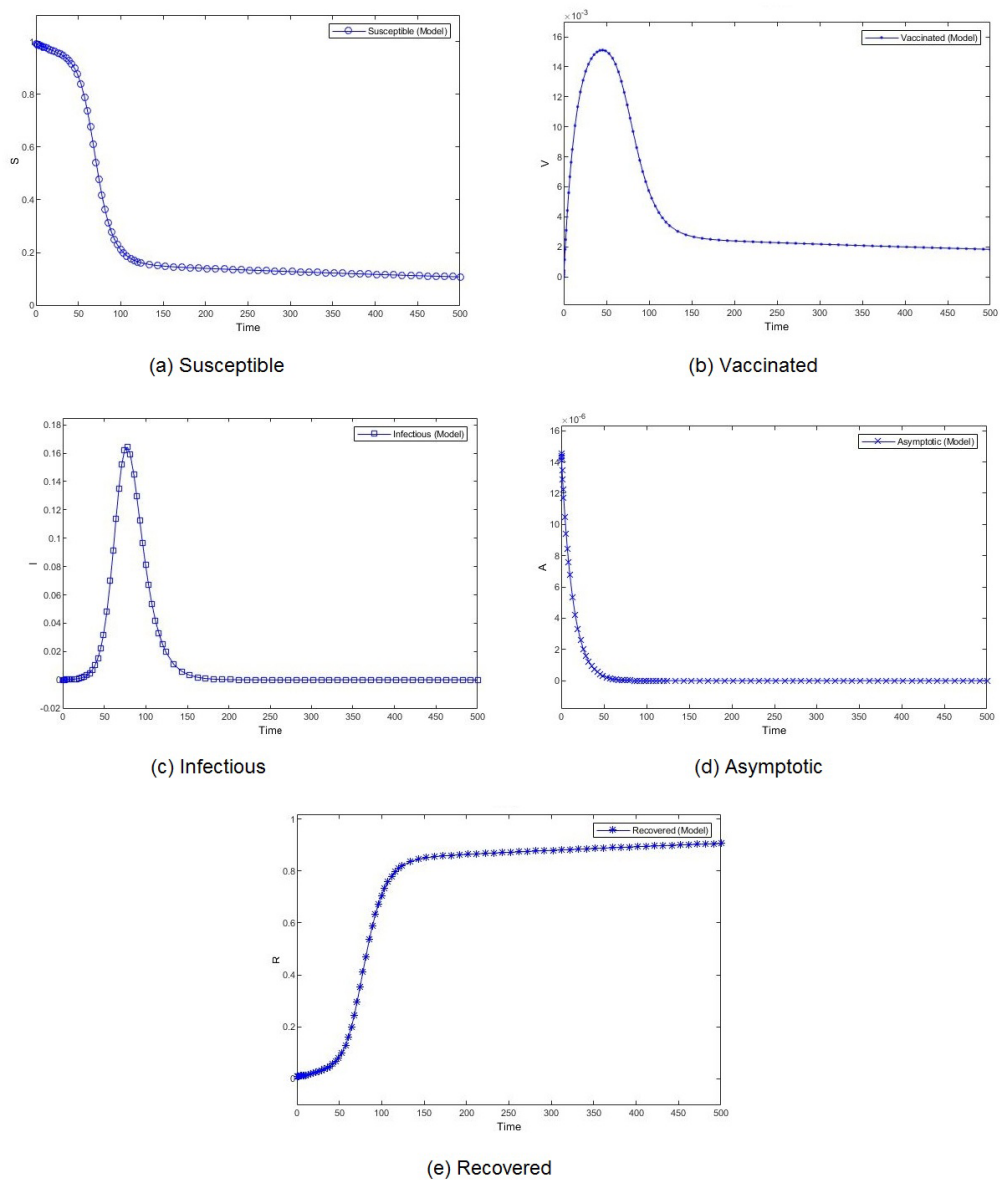
Percentage of susceptible (A), vaccinated (B), infectious (C), asymptomatic (D), and recovered (E) individuals. *β* = 0.233, *ψ* = 0.0012.

**Figure 5:**
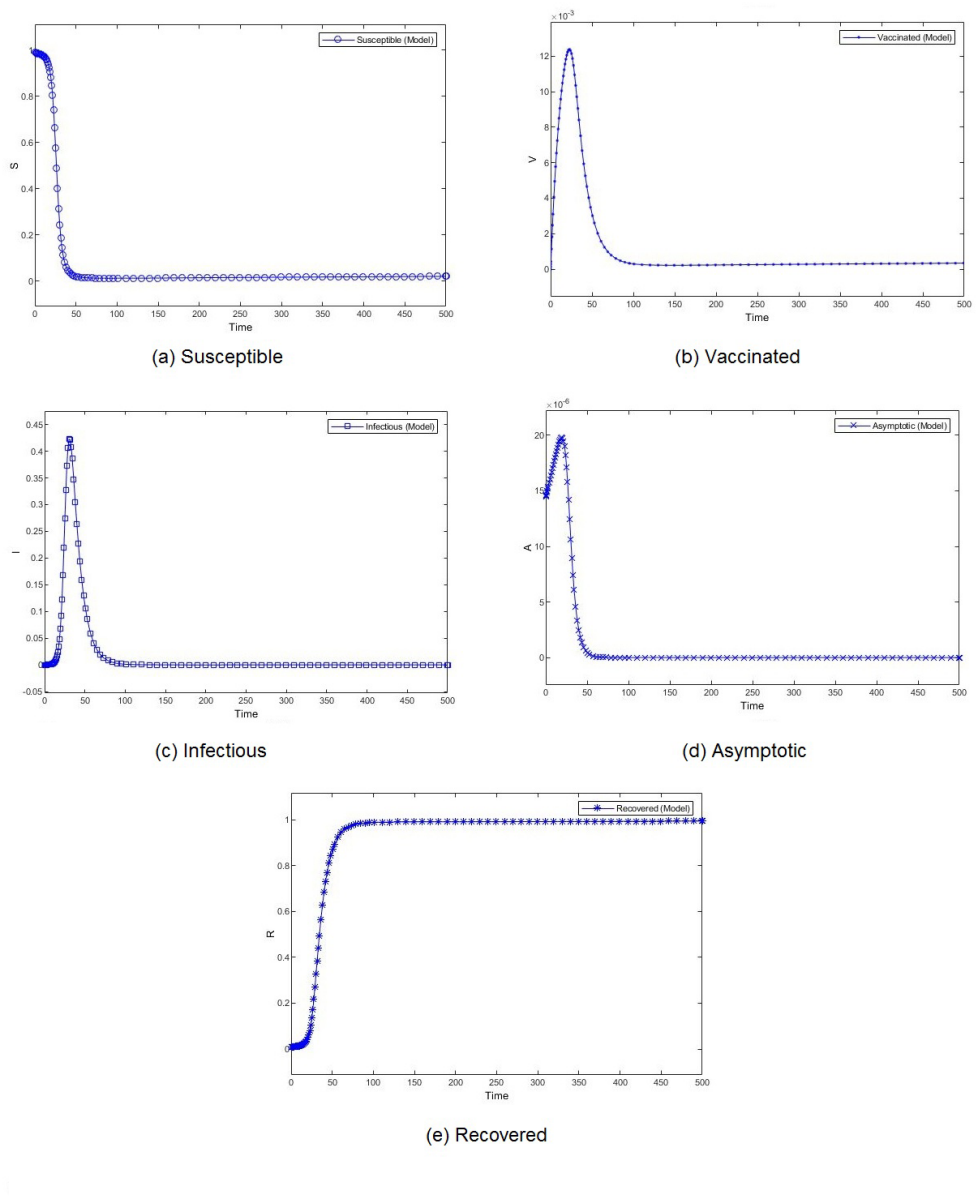
Percentage of susceptible (A), vaccinated (B), infectious (C), asymptomatic (D), and recovered (E) individuals. *β* = 0.462, *ψ* = 0.0012.

Figure 3 shows the weekly number of new active cases and recovered after infection in KSA, starting from 18/12/2020, the date when the vaccination starts. We can see that after 12 weeks, the number of cases raises. This behaviour was surprising for a population waiting to see the effect of vaccination. It’s only after 31 weeks that the number of new cases start to decrease. The same phenomena was observed in both Figs. 4C and 5C. The theatrical results is conform to actual statistics. The effect of vaccination is not immediate; it needs several weeks to observe a decrease in the number of new infectious cases.

We compare the evolution in time of the five compartments (S, V, I, A, and R) presented in our model, for two different transmission rate and with the Saudi vaccination rate. With different dynamics at the beginning, both scenarios show a convergence to a stable state. We observe almost similar patterns for S, V. I and R. The number of susceptible individuals *S* decreases slowly at the beginning and then, we observe a drastic decline. Obviously, the number of recovered follows the same slow and then fast pace but in decrease. The number of vaccinated individuals *V* increases gradually at the beginning and then it begins to fall down. The number of infectious individuals *I* remain stable for a short period to witness an expansion followed by a decline. The number of asymptotic individuals show different evolution patterns for two considered scenarios. When we set a low value for the virus transmission rate, this number immediately shrinks. However, when we set a high value for the virus transmission rate, this number increases before shrinking.

The effect of the transmission rate can also be observed in the amplitude of each category of individuals. When the models converge, the number of infectious and asymptotic individuals are zero. We emphasize here our theoretical result, mentioned in Theorem 1, that states that if both *R*_1_ and *R*_2_ are less than one, the disease free equilibrium is stable. This result is consistent with the simulation results. The difference between the two considered scenarios lies in the percentage of susceptible and vaccinated individuals in the equilibrium. This percentage is very low when the transmission rate is high. Although the percentages of vaccinated individuals are close, we observe a remarkable difference in the number of infectious individuals. When the transmission rate is high, almost 40% of the population is infected, which rises public health issues.

### Managerial insights and practical implications

By March 2022, several countries, including Saudi Arabia, have simplified COVID-19 rules by relaxing some safe management measures such as social distancing and mask wearing. However, the Saudi ministry of health kept the condition of three vaccine doses. This decision is part of a public health strategy that continues to monitor virus variants, mainly Omicron and its sublineages BA.1 and BA.2. WHO’s Technical Advisory Group requests countries to continue to be vigilant because of potential significant rise in the number of infections. Although these variants might resist neutralizing antibodies in the blood, the vaccine allows preventing severe illness and death. Unlike several countries, Saudi Arabia was spared from the lack of oxygen generators, the most important medical equipment for hospitalized patients. The main concern is, however, regulations regarding vaccination. The vaccine was made mandatory for individuals over 12 years old. The open question is the need for vaccine for children aged from 5 to 11 years. Therefore, managers need to simulate the effect of vaccination and make predictions of the virus spread. This work tries to provide them with an efficient tool that captures the specificity of the Saudi case.

## Conclusion

In this article, we present a mathematical model for COVID-19, based on the virus behaviour. Our main target is to evaluate the effect of vaccination on the population. The presence of individuals presenting no symptoms and the immunity loss are the main characteristics that make this virus different from other known and already modeled diseases. We provide analytical expression of the effective reproduction number with is a key factor to determine necessary conditions for endemic and disease free equilibrium. We supported our theoretical findings with the numerical analysis applied to the Saudi case. The main findings of this work are as follows.

• People can observe that the vaccine helps to reduce the severity of symptoms. We gave a mathematical proof that the vaccine reduces the transmission rate.
• The vaccination campaign in Saudi Arabia was immediately followed by the rise in the number of infections. We have showed that this observation is mathematically justified and this rise is a necessary transition before the increase of new infections.
• By adjusting the model parameters based on collected data, we provide the decision-makers with the vaccination rate necessary for virus spread control.

Recently, the scientific community is observing the new variants that show each time different patterns. We aim, in the future, to develop a new model with the new observed characteristics of variants such as beta and omicron. As discussed in the previous section, several countries including Saudi Arabia did not face medical resource shortages. However, as highlighted by Lotfi et al., (2021), the management of medical waste is critical during COVID-19 pandemic. Moreover, the number of asymptomatic and untested symptomatic infections is uncertain. As future work, we propose to capture disease dynamic uncertainty and incorporate risk assessment, to alleviate the impacts of pandemic peak. Hybrid fuzzy, data-driven, robust optimization, and stochastics (Lotfi et al., 2022a; Lotfi et al., 2022b) are examples of uncertainty analysis methods. Conditional value at risk (CVaR), which is the average shortfall beyond the VaR point, is a consistent and coherent risk assessment measure.

## Supplemental Information

10.7717/peerj-cs.959/supp-1 Supplemental Information 1 Code

## Additional Information and Declarations

### Data Availability

The following information was supplied regarding data availability: The data is available at KAPSARC’s Data Portal: Saudi Arabia Coronavirus disease (COVID-19) situation:
https://datasource.kapsarc.org/explore/dataset/saudi-arabia-coronavirus-disease-covid-19-situation/table/?disjunctive.daily_cumulative&disjunctive.indicator&disjunctive.event&disjunctive.city_en&disjunctive.region_en&sort=date&fbclid=IwAR3Ybv-hqe0J8IXoXmMwY4LBCkoI7wuvgyP4JgcpJyM6V77tzBUUnh53dhU.
